# Validation of Perfusion Quantification with 3D Gradient Echo Dynamic Contrast-Enhanced Magnetic Resonance Imaging Using a Blood Pool Contrast Agent in Skeletal Swine Muscle

**DOI:** 10.1371/journal.pone.0128060

**Published:** 2015-06-10

**Authors:** Stefan Hindel, Anika Sauerbrey, Marc Maaß, Stefan Maderwald, Marc Schlamann, Lutz Lüdemann

**Affiliations:** 1 Department of Radiotherapy, Medical Physics, University Hospital Essen, Essen, North Rhine-Westphalia, Germany; 2 Department of General and Visceral Surgery at Evangelical Hospital Wesel, Wesel, North Rhine-Westphalia, Germany; 3 Erwin L. Hahn Institute for Magnetic Resonance Imaging, Essen, North Rhine-Westphalia, Germany; 4 Department of Diagnostic and Interventional Radiology and Neuroradiology, University Hospital Essen, Essen, North Rhine-Westphalia, Germany; West German Cancer Center, GERMANY

## Abstract

The purpose of our study was to validate perfusion quantification in a low-perfused tissue by dynamic contrast-enhanced magnetic resonance imaging (DCE-MRI) with shared k-space sampling using a blood pool contrast agent. Perfusion measurements were performed in a total of seven female pigs. An ultrasonic Doppler probe was attached to the right femoral artery to determine total flow in the hind leg musculature. The femoral artery was catheterized for continuous local administration of adenosine to increase blood flow up to four times the baseline level. Three different stable perfusion levels were induced. The MR protocol included a 3D gradient-echo sequence with a temporal resolution of approximately 1.5 seconds. Before each dynamic sequence, static MR images were acquired with flip angles of 5°, 10°, 20°, and 30°. Both static and dynamic images were used to generate relaxation rate and baseline magnetization maps with a flip angle method. 0.1 mL/kg body weight of blood pool contrast medium was injected via a central venous catheter at a flow rate of 5 mL/s. The right hind leg was segmented in 3D into medial, cranial, lateral, and pelvic thigh muscles, lower leg, bones, skin, and fat. The arterial input function (AIF) was measured in the aorta. Perfusion of the different anatomic regions was calculated using a one- and a two-compartment model with delay- and dispersion-corrected AIFs. The F-test for model comparison was used to decide whether to use the results of the one- or two-compartment model fit. Total flow was calculated by integrating volume-weighted perfusion values over the whole measured region. The resulting values of delay, dispersion, blood volume, mean transit time, and flow were all in physiologically and physically reasonable ranges. In 107 of 160 ROIs, the blood signal was separated, using a two-compartment model, into a capillary and an arteriolar signal contribution, decided by the F-test. Overall flow in hind leg muscles, as measured by the ultrasound probe, highly correlated with total flow determined by MRI, *R* = 0.89 and *P* = 10^−7^. Linear regression yielded a slope of 1.2 and a y-axis intercept of 259 mL/min. The mean total volume of the investigated muscle tissue corresponds to an offset perfusion of 4.7mL/(min ⋅ 100cm^3^). The DCE-MRI technique presented here uses a blood pool contrast medium in combination with a two-compartment tracer kinetic model and allows absolute quantification of low-perfused non-cerebral organs such as muscles.

## Introduction

Solid tumors are characterized by a heterogeneous distribution of blood flow with cells that are located far from a functional blood vessel having significantly lower oxygen concentrations than normal tissue [1]. This hypoxia represents a significant barrier to effective radiotherapy. Since hypoxic tumor cells remain clonogenic, their resistance to treatment strongly influences the outcome of radiotherapy [2]. Tumors also often show acidity in low-flow regions. Hypoxic-acidic regions may stimulate progression to a more metastatic phenotype. On the other hand, in normal tissues, hypoxia and acidity also induce angiogenesis, which is expected to improve perfusion [3, 4].

Worldwide, breast cancer is the most common cancer in women, accounting for 25% of all cases [5]. Almost invariably, human breast tumors contain regions of hypoxia [1], and 90% of breast cancer deaths are the result of metastasis [1, 6]. Prostate cancer is the second most common cause of cancer and the fifth leading cause of cancer-related death in men worldwide [7]. Hypoxia may be a reason why even optimal radiochemotherapy often fails to achieve local control of advanced head and neck cancer [8, 9]. Hypoxia is also a typical feature of prostate cancer [10]. It has been shown that local dose escalation may improve the outcome of radiotherapy in patients with hypoxic prostate cancer [11]. The proportion of hypoxic tissue is inherently coupled to oxygen supply or tissue perfusion. The degree of perfusion allows to quantify the hypoxic tissue fraction [12].

Assuming the density of mammalian skeletal muscle tissue to be about 1.06 g/mL [13] Wilson et al. determined perfusion in the breast to be around 30 mL/(min⋅100cm^3^) [14]. Average perfusion in normal prostate tissue is 22.2 mL/(min⋅100cm^3^) vs. 28.8 mL/(min⋅100cm^3^) in benign prostate hyperplasia [10]. Perfusion in prostate cancer is approximately three times higher than in normal prostate tissue (mean: 60 mL/(min⋅100cm^3^)) and is subject to pronounced intra- and inter-tumor variability [10]. Most reviews on skeletal muscle blood flow indicate that maximum perfusion in mammalian skeletal muscle is in the range of 48 mL/(s⋅cm^3^)–480 mL/(min⋅100cm^3^) [15]. Perfusion in normal mammalian skeletal muscle is approx. 1 to 21 mL/(min⋅100cm^3^) [16]. To use dose painting for prescribing a nonuniform radiation dose to a tumor, it is necessary to quantitatively determine tumor oxygenation with high spatial resolution using imaging techniques.

With its excellent soft tissue contrast and high spatial resolution, magnetic resonance imaging (MRI) reliably distinguishes different soft tissues from each other. Additionally, dynamic contrast-enhanced MRI (DCI-MRI) provides information on tumor perfusion. So far, however, dynamic MRI has mainly been used for quantifying perfusion in highly perfused organs such as the heart, the brain, the kidneys, and the liver. Particularly quantification of blood flow in the highly perfused brain is easier to accomplish because the blood-brain barrier eliminates problems such as truncation errors caused by extravasation of the clinically widely used low-molecular-weight contrast agents. Sourbron and Buckley [17] suggest that perfusion quantification in nonbrain tissues is easier to accomplish with use of intravascular indicators. To the best of our knowledge, there is only one DCE-MRI study that uses an non-imaging technique to validate blood flow in highly-perfused tissue [18].

In areas of low perfusion, more parameters including delay and dispersion may affect perfusion estimates compared with highly perfused tissues [19–23]. Since the use of an intravascular tracer instead of a low-molecular-weight tracer reduces the number of model parameters necessary to assess local perfusion, a more reliable noninvasive method for absolute quantification of blood flow in low-perfusion tissues can be established if an intravascular contrast medium (CM) is used.

The variability of tissue perfusion underlines the importance of validating methods for blood flow quantification at different flow levels and to specifically test such methods in low-flow organs and tissues. In the present study, the skeletal hind leg musculature of swine was used as a general model for low-flow tissues. Thus, the aim of the present study is to suggest a highly accurate method for measuring perfusion minimally invasively in low-perfusion organs such as breast, prostate, and skeletal muscle. To the best of our knowledge, this is the first DCE-MRI perfusion validation study that uses an independent nonimaging-based technique for comparison in a low-perfused tissue.

## Materials and Methods

### Study Subjects

To provide a realistic setting for clinical applications we chose pigs of similar body size as humans. A total of 13 adult pigs were sacrificed for our study (German Landrace or hybrid form; age, approximately 20 weeks; body weight, 56 to 67 kg; no food restriction). In six pigs, the surgical technique and the adenosine dose (Adenosin Life Medical, 5 mg/mL, Carinopharm, Germany) response for flow enhancement were established and optimized, and the MRI sequences and the acquisition protocol were optimized for perfusion and anatomic imaging. In these preliminary experiments, we identified the adenosine doses needed to achieve three different target levels of stable perfusion. These adenosine doses were set individually for each pig and ranged from 0.3 to 6.25 *μ*Mol/min. In seven pigs, flow measurements were carried out in the MRI scanner. The pigs were not fed the night before the study. For more details about the surgical technique and the adenosine dose response see [24]. The animal study was approved by the Landesamt für Natur, Umwelt und Verbraucherschutz Nordrhein-Westfalen (approval number: 84-02.04.2012.A208) and all experiments were performed in accordance with the regulations of the German animal protection law. At the end of each experiment, the pigs were sacrificed under a higher level of anesthesia by the injection of T61 (0.3 mL/kg).

### Surgical Technique

Premedication of the pigs was done by intramuscular injection of 30 mg/kg ketamine (ketamine 10%, Ceva Tiergesundheit GmbH, Germany) for alleviation of potential pain, 2 mg/kg azaperone (Stresnil Janssen-Cilag GmbH, Germany) for suppression of nausea and for sedation, and 0.02–0.05 mg/kg atropine sulfate (Atropinsulfat, B. Braun Melsungen AG, Germany) for reduction of salivation, inhibition of gastrointestinal activity, and for relaxation of smooth muscles.

**Fig 1:**
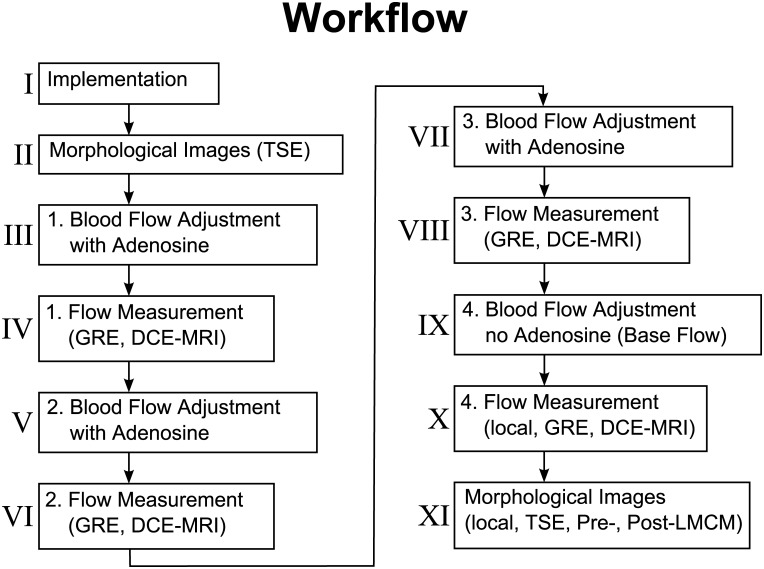
Exemplary workflow for perfusion measurement in a single swine. Exemplary workflow for perfusion measurement in a single swine.

The experimental workflow is presented in Fig 1. About thirty minutes after premedication, a peripheral 20-G venous catheter was placed in an ear vein to start total intravenous (IV) anesthesia. For this purpose, an infusion pump was used to inject 4–7 mg/kg/h propofol (Propofol-ratiopharm, Ratiopharm, Germany) for regulated anesthesia, 0.1–0.5 mg/kg/h midazolam (midazolam injection solution 0.5%, Germany) for anxiety reduction, central muscle relaxation, and sedation, and 0.0015 mg/kg/h fentanyl (fentanyl citrate solution 3.925 mL/50 mL, Germany) for pain relief. A tracheal tube was inserted into the pig’s throat (Hi-Contour cuffed tracheal tube, ID 8.0, Mallinckrodt, Ireland), and the pig was ventilated with a respiratory device (Fabius, Draeger, Germany).

To create a setting most similar to the potential clinical application in human patients, a central venous catheter (3-Lumen-ZVK-Set, ARROWg+ ard Blue, Arrow, Germany) was placed in the jugular vein on the right side of the neck for systemic CM administration (see Fig 1.I). Subsequently one of the femoral arteries was exposed, preferably the right one. The femoral artery was catheterized proximally using the Seldinger technique to enable local administration of adenosine or CM. The metal-free, MRI-compatible catheter (Arterial Leader Cath, Vygon, France) was advanced proximally and fixed with several sutures. Distal to the catheter, an ultrasound (US) flow probe (T206, Transonic Systems Inc., Ithaca, NY) was implanted around the femoral artery to measure blood flow invasively. The US probe was also fixed with sutures. The cable of the probe was led out of the wound in a direct way by tunneling of the subcutaneous tissue [24].

### MRI Technique

Heart rate and oxygen saturation were monitored with an MRI-compatible monitoring device (Veris, Medrad, Germany). The pig was placed in the supine position on the scanner table. The table had an integrated 32-channel spine coil (Siemens Magnetom Aera 1.5 T, Siemens Healthcare, Erlangen, Germany). A surface coil (Tim body coil, 18 RF channels, Siemens Healthcare, Erlangen, Germany) was placed on the hind limbs. The blood flow measurement data from the US flow probe were continuously recorded using LabVIEW 2012 (National Instruments, Austin, Texas, USA), an A/D converter card (NI USB-6211, National Instruments, Austin, Texas, USA), and a standard netbook with Microsoft Windows XP (Microsoft, Redmond, Washington, USA).

After implantation of the catheters and the Doppler flow probe, morphological images were acquired before CM administration (Figs 1.II and 2). To induce vasodilatation, an infusion pump was used to locally inject adenosine via the Seldinger catheter in the femoral artery. To achieve constant blood flow levels, the adenosine perfusion rate was individually adapted in all pigs during a perfusion measurement (Fig 1.III). Three perfusion measurements at different blood flow levels were performed per animal. To avoid systematic errors, the order of flow levels following adenosine administration was randomly changed. This was done to compensate for systematic errors due to signal saturation resulting from CM accumulation. After blood flow adjustment, the MRI blood flow measurement routine was performed (Fig 1.IV). Steady blood flow occurred after approximately five minutes. Two further flow adjustments and flow measurements with systemic CM administration were performed (Fig 1.V–1.VIII), followed by a perfusion measurement with local CM administration (Fig 1.IX and 1.X). After the perfusion measurements, the supply area of the femoral artery was determined by locally injecting CM into the femoral artery. Morphological turbo spin echo (TSE) and dynamic gradient echo (GRE) images were used to generate difference images (Figs 1.IX–1.XI and 2). The difference images were created by subtraction of the pre-CM images from the post CM-images. They were necessary to separate the region supplied by the femoral artery (where the adenosine-induced increase in blood flow takes place) from the rest of the organism.

**Fig 2:**
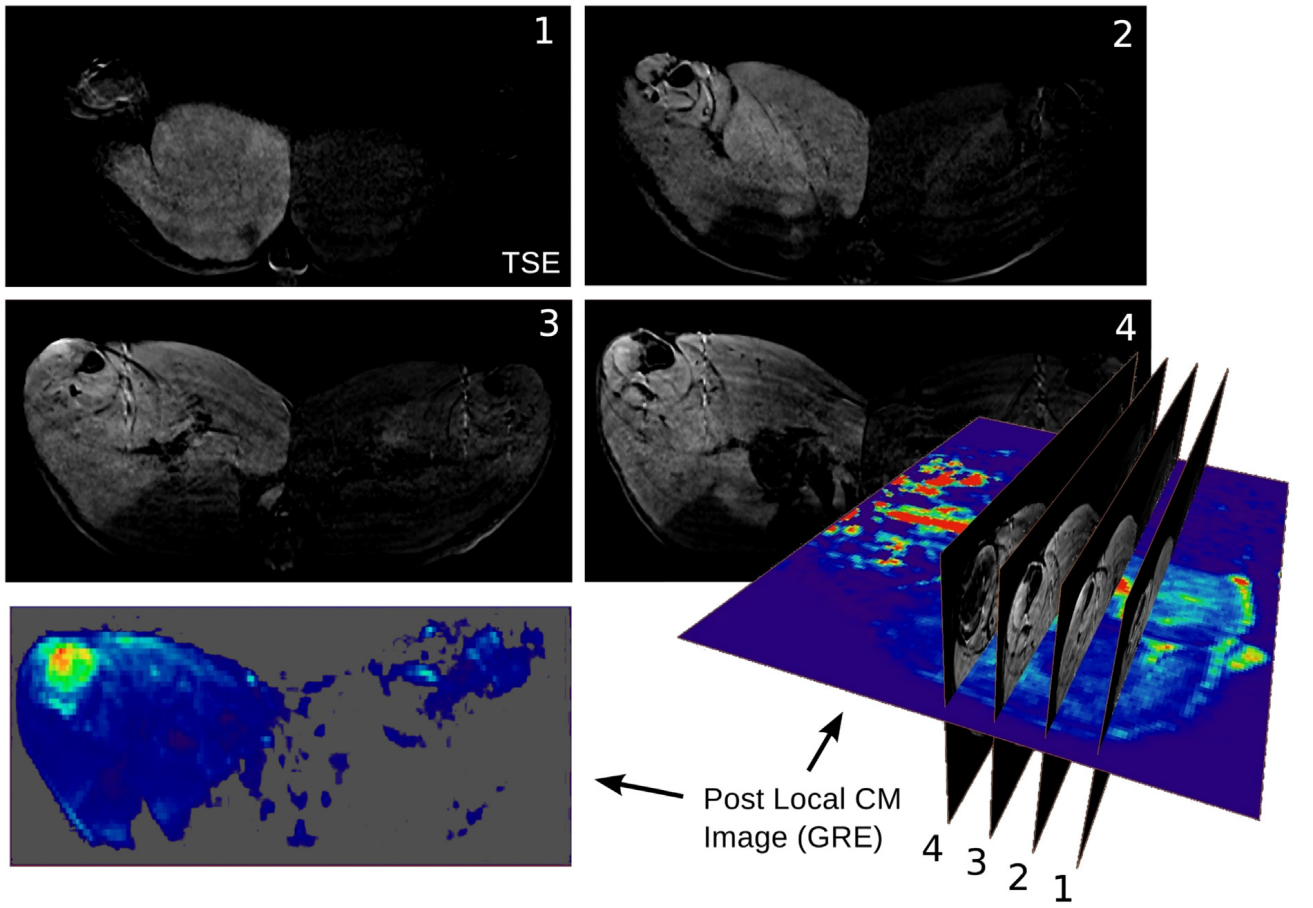
Pre- and post-LMCM difference images. Pre- and post-LMCM difference images. T1-weighted TSE and GRE images were acquired before and after local injection of contrast agent into the right femoral artery. There is significant brightening of the area supplied by the femoral artery (represented here on the left side of the axial MR images of the pig’s abdomen).

The non-CM-enhanced T1-weighted morphological images with and without fat suppression were also acquired for direct orientation during the experiment (Fig 1.II). The protocol included an axial T1-weighted TSE sequence with fat suppression in the transverse plane that was acquired with the following parameters: repetition time, TR = 625 ms, echo time, TE = 12 ms, flip angle, *α* = 150°, and voxel size, 0.9 × 0.9 × 7.0mm^3^.

The perfusion protocols (Fig 1.IV, 1.VI, 1.VIII and 1.X) included 3D GRE sequences (TWIST, Siemens Healthcare, Erlangen, Germany) for estimation of baseline magnetization and relaxation rate using *α* = 5°, 10°, 20°, and 30° and the following parameters: TR = 2.69 ms, TE = 0.86 ms, voxel size of 2.9 × 2.9 × 4.5mm^3^, 160 × 128 × 48 reconstruction matrix, frequency encoding in the axial direction, parallel imaging with GRAPPA with 32 central k-space lines and an acceleration factor of 6; central k-space region A was 100%. The 3D gradient echoes with multiple flip angles were measured before each CM injection. With identical parameters, dynamic imaging was performed for 100 measurements with a high temporal resolution of approximately 1.5 s with identical parameters, except for *α* = 30° and the use of the shared k-space. The central 20% of the central k-space lines were scanned every time. The peripheral 80% of the k-space lines were split, and each line was sampled every fifth acquisition. After the fifth acquisition of the first dynamic sequence, 0.1 mL/kg body weight of blood pool contrast medium (BPCM) (0.25 mMol/mL gadofosveset trisodium, Vasovist Bayer Schering, Berlin, Germany/Ablavar, Lantheus Medical Imaging, Inc., USA) was injected via the central venous catheter at a flow rate of 5 mL/s. CM administration was followed by injection of 20 mL 0.9% saline solution at the same rate. Time-dependent maps of T1 relaxation rate changes were computed using the method of Li et al. [25].

Increased or reduced blood flow was induced by local injection of adenosine (Adenosin Life Medical, 5 mg/mL, Carinopharm, Germany) into the femoral artery using a syringe pump. The dose was chosen according to its flow-enhancing effect. After about 5 min, a steady blood flow, measured by the US flow probe, was achieved, and MRI measurement was started. A total of up to three perfusion measurements were performed per animal. To avoid systematic errors, the order of flow levels following adenosine administration was randomly changed. This was done to compensate for systematic errors due to signal saturation resulting from CM accumulation.

The flow measurement protocols were followed by acquisition of anatomical images without CM administration (Fig 1.XI). Then scans with rapidly extravasating LMCM were obtained. A dose of 2 mL of LMCM was administered locally at 1.0 mL/s.

### Volumetry

**Fig 3:**
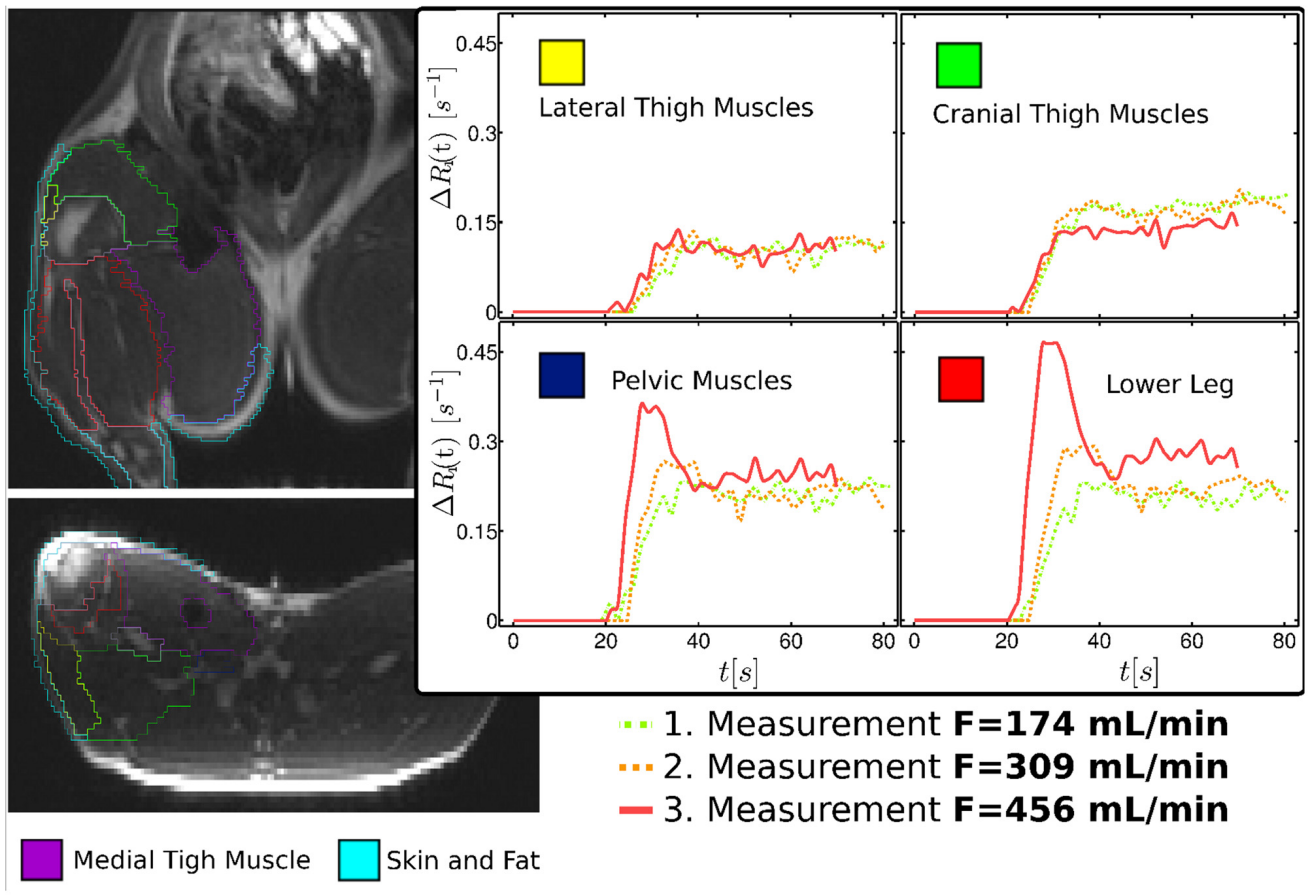
Muscle segmentation and examples of tissue blood curves. Muscle segmentation and examples of tissue blood curves obtained by averaging over all segment voxels. The adenosine response and thus the flow increase were inhomogeneously distributed over the different muscle segments. The dash-dot line represents the measurement of the first flow state in which a flow of 174 mL/min was measured at the femoral artery with the Doppler flow probe. The dashed (solid) line represents the second (third) measurement with a Doppler flow of 309 (456) mL/min.

Volumetry, segmentation (Fig 3), and determination of muscle perfusion were performed using the AmiraDev 5.2 visualization package (Mercury Computer Systems, Berlin, Germany) on a Debian Linux 64 bit workstation. The functionality of AmiraDev 5.2 has been extended by the application of AmiraDev tools on dynamically acquired data by software packages implemented as dynamic link libraries in AmiraDev.

The morphological MR images and pre and post local LMCM difference images (T1-TSE) (Fig 1.II and 1.XI) and the dynamic GRE images with local CM administration (Fig 1.IX and 1.X) were used for separate segmentation of eight different tissue regions (Fig 3), henceforth called segments: medial, cranial, lateral, and pelvic thigh muscles, lower leg, bones, skin, and fat. Furthermore, the percentage volume of each tissue compartment separated in this way relative to the total volume of the hind leg was calculated.

### Perfusion Evaluation

The baseline dynamic scans were used for calculation of baseline 3D longitudinal relaxation rates (*R*_10_) and magnetization maps (*M*_0_). The relaxation rate (*R*_10_) and magnetization (*M*_0_) maps were used to convert the dynamic k-space-sampled GRE scans into 4D relaxation rate change maps *R*_1_(*t*). The relaxation change-time curves are directly proportional to the concentration-time curves via the expression [25]:

$$\begin{array}{c} C\left(t\right)=\frac{\Delta R}{{ℜ}_{1}}, \end{array}$$ (1)

where Δ*R* is the relaxation rate change and ℜ_1_ = 19 L ⋅ mmol^−1^⋅*s* ^−1^ (at 1.5T and 37°C) is the relaxivity of gadofosveset [26]. To calculate perfusion, the arterial input function (AIF) is necessary. The AIF, $C_{A}^{\left(est\right)}(t)$, was extracted from the voxels that were definitely and completely localized in the abdominal aorta in order to avoid partial volume effects. The volume of interest (VOI) of the AIF consisted of approximately 10 voxels, covering an absolute volume of 378 mm^3^. The tissue curves of the eight segments and the AIF were extracted from the dynamic relaxation change map.

The first 60 seconds of the relaxation rate change-time curves of the averaged voxels of the AIF and tissue segments were used for model fitting. Before numerical analysis of the time curves, the temporal resolution of the curves was increased to 0.1 seconds using linear interpolation. Perfusion was calculated by numerical deconvolution of the tissue relaxation rate change curves with noninvasively determined arterial input curves using an analytical approach for the residue function as described in detail by Østergaard et al in [27].

**Fig 4:**
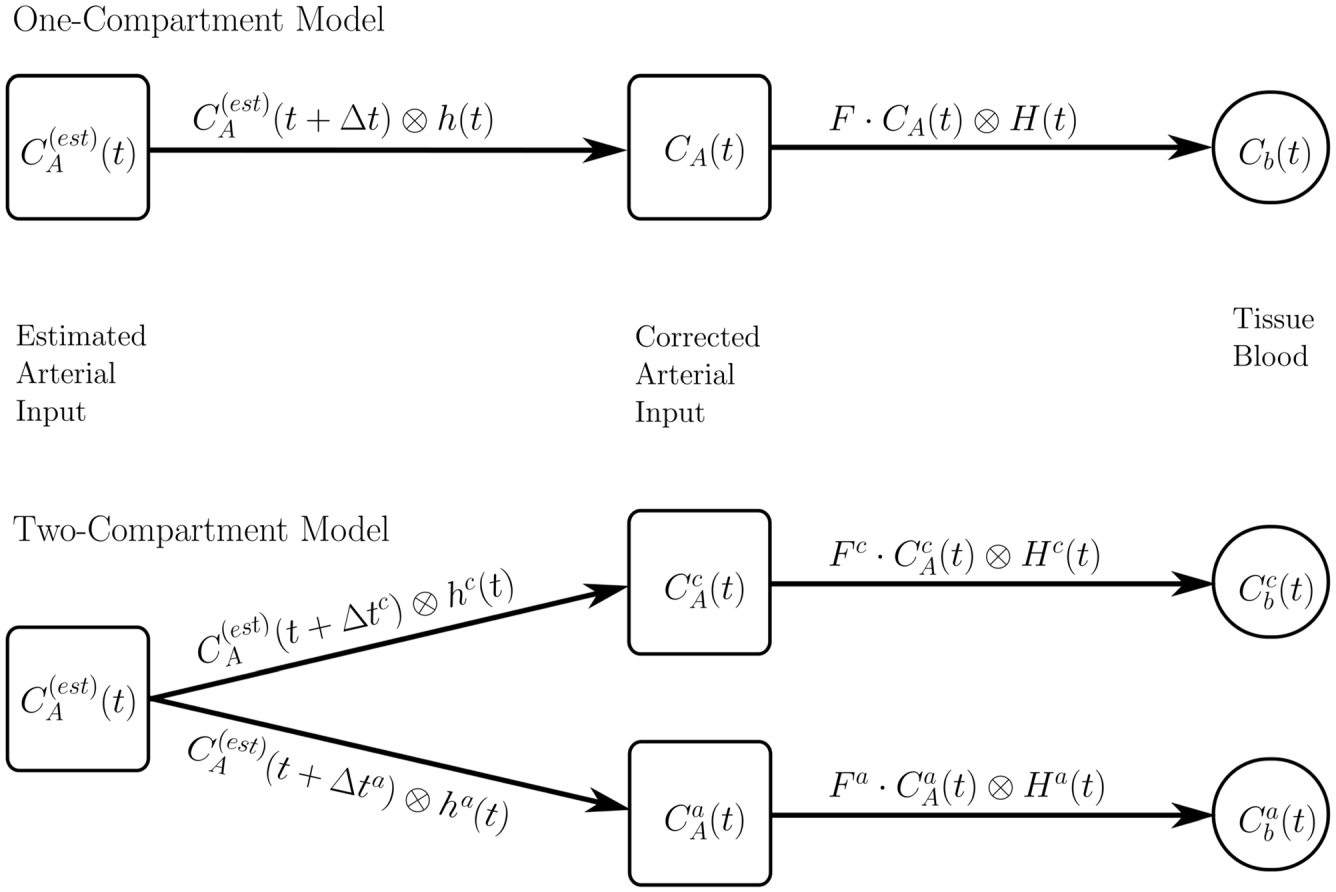
Diagrams of the one- and two-compartment model. Diagrams of the one- and two-compartment model.

The design of our models (depicted schematically in Fig 4) was based on the indicator dilution theory and required the deconvolution of the tissue blood concentration-time curve in tissue capillaries and tissue arterioles, $C_{b}^{i}(t)$ [28]:

$$\begin{array}{c} C_{b}^{i}\left(t\right)=F^{i}\cdot C_{A}^{i}\left(t\right)⊗H^{i}\left(t\right), \end{array}$$ (2)

where *i* = *a*, *c* indicates the respective compartment (arteriolar or capillary), *F* ^*i*^ is perfusion, and *H* ^*i*^(*t*) are the respective residue functions with mean transit times *T* ^*i*^:

$$\begin{array}{c} H^{i}\left(t\right)=e^{-t/T^{i}}. \end{array}$$ (3)

A single exponential was used as a first-order model to describe the residue function. For such a system, the residue function is an exponential [29]. We used general least-squared minimization to fit for *T* ^*i*^ and *F* ^*i*^ [27].

To calculate the AIFs $C_{A}^{i}(t)$ for the two single compartments we corrected $C_{A}^{\left(est\right)}(t)$ for delay Δ*t* ^*i*^ and dispersion times 1/*β* ^*i*^:

$$\begin{array}{c} C_{A}^{i}\left(t\right)=C_{A}^{\left(est\right)}\left(t+\Delta t^{i}\right)⊗h^{i}\left(t\right), \end{array}$$ (4)

where

$$\begin{array}{c} h^{i}\left(t\right)=\beta^{i}\cdot e^{-\beta^{i}t} \end{array}$$ (5)

are the vascular transport functions that describe bolus dispersion during effective transit times 1/*β* ^*i*^ from the site of AIF measurement to the input to the particular region of interest (ROI).

**Fig 5:**
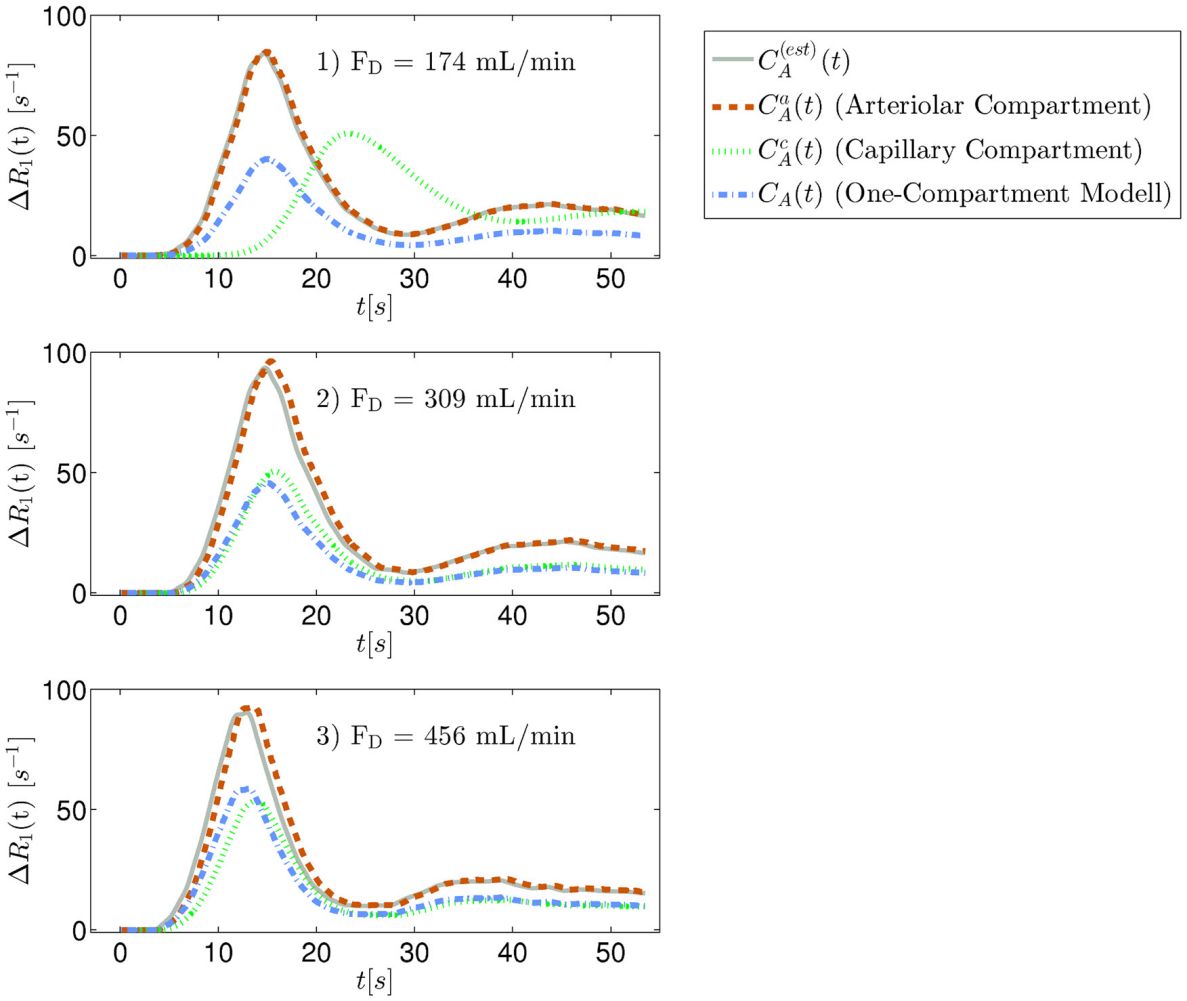
Examples of correction of the arterial input function (AIF). Examples of correction of the arterial input function (AIF). The case in question is for the lower leg and belongs to the blood-tissue curves from the example presented in the Results section.

Fig 5 shows the AIFs (gray solid line) measured in the aorta for the three flow measurements of the same experiments as in the example discussed in the Results section. In addition, the corrected AIFs for the single-compartment model and for the capillary and arteriolar compartment of the two-compartment model are shown.

Finally, the sum

$$\begin{array}{c} C_{b}\left(t\right)=v_{b}^{c}C_{b}^{c}\left(t\right)+v_{b}^{a}C_{b}^{a}\left(t\right) \end{array}$$ (6)

gives the average tissue concentration time-curve in the ROI under consideration, where $v_{b}^{c}$ and $v_{c}^{a}$ are the fractional capillary and arteriolar blood volume, respectively.

The MRI relaxation change-time curve for each segment was determined by averaging the contributions of all voxels over the volume of the entire segment. Within these ROIs, mean medullary and cortical perfusion was determined in mL/min/100cm^3^ via model fitting with the one- and two-compartment model. As proposed by Sourbron et al. [30] and Donaldson et al. [31], the F-test for model comparison was used to decide whether to use the results of the one-compartment or two-compartment model fit.

The segment volume was multiplied with the perfusion of the respective segment to determine flow in each segment. The flow probe is surrounded by an artifact area with a radius of approximately 1 cm, which was excluded from flow calculation. Total flow was calculated by summing up the contributions of the individual segments. The total flow values were compared with the invasive blood flow measurements provided by the implanted US flow probe.

The full protocol with perfusion measurements at three different blood flow levels was performed in six of the seven pigs, while only two measurements were performed in one pig (Fig 1.III–1.VIII). For the flow measurements in each pig, different flow levels were aimed at by adenosine administration. To minimize a possible effect of CA accumulation, the order in which the three different flow levels were induced was varied, i.e. the order of flow levels was varied from highest to lowest in part of the pigs and vice versa in the other pigs.

The tissue blood flow curves were generated by averaging large tissue regions located in the center of the area covered by the body coil. The AIF, however, was derived from an area of approximately 10 voxels in the aorta close to the coil edge. This method made it necessary to correct the model flow values with regard to the AIF steady state phase of the individual measurements. For each of the *n* = 20 flow measurements, we calculated the time-dependent mean AIF relaxation rate *w*_*n*_ by averaging all AIF time curves over 20 seconds starting approximately 60 seconds after bolus arrival for each of the n measurements:

$$\begin{array}{c} w_{n}=<Ca\left(t_{i}\right){|}_{i=j,...,k}>,n=1...20, \end{array}$$ (7)

where *i* = 0 is the index of the time of bolus arrival, *j* is the index for the time increment 60 seconds after bolus arrival, and *k* is the time increment 20 seconds after *t*_*j*_. Then we calculated the mean AIF amplitude

$$\begin{array}{c} W=<w_{n}> \end{array}$$ (8)

and calculated a normalized AIF for each flow measurement by dividing the individual AIF curve height by the overall mean AIF curve height:

$$\begin{array}{c} F_{n}^{\text{Norm}}=c_{n}\cdot F_{n}, \end{array}$$ (9)

with

$$\begin{array}{c} c_{n}=w_{n}/W. \end{array}$$ (10)

### Statistics

Statistical analysis was performed using Microsoft Excel 2010 (Microsoft, Redmond, Washington, USA) and Matlab R2011b (The MathWorks Inc., Natick, Massachusetts, USA). All values were expressed as means and standard deviations as error. The correlation between the two models (invasive flow measurement and DCE data) was assumed to be linear, and the error between the methods was assumed to be normally distributed. Correlations were calculated using the method of linear regression analysis, and significance was estimated by the Pearson correlation coefficient. *P* < 0.05 was considered significant.

## Results

**Fig 6:**
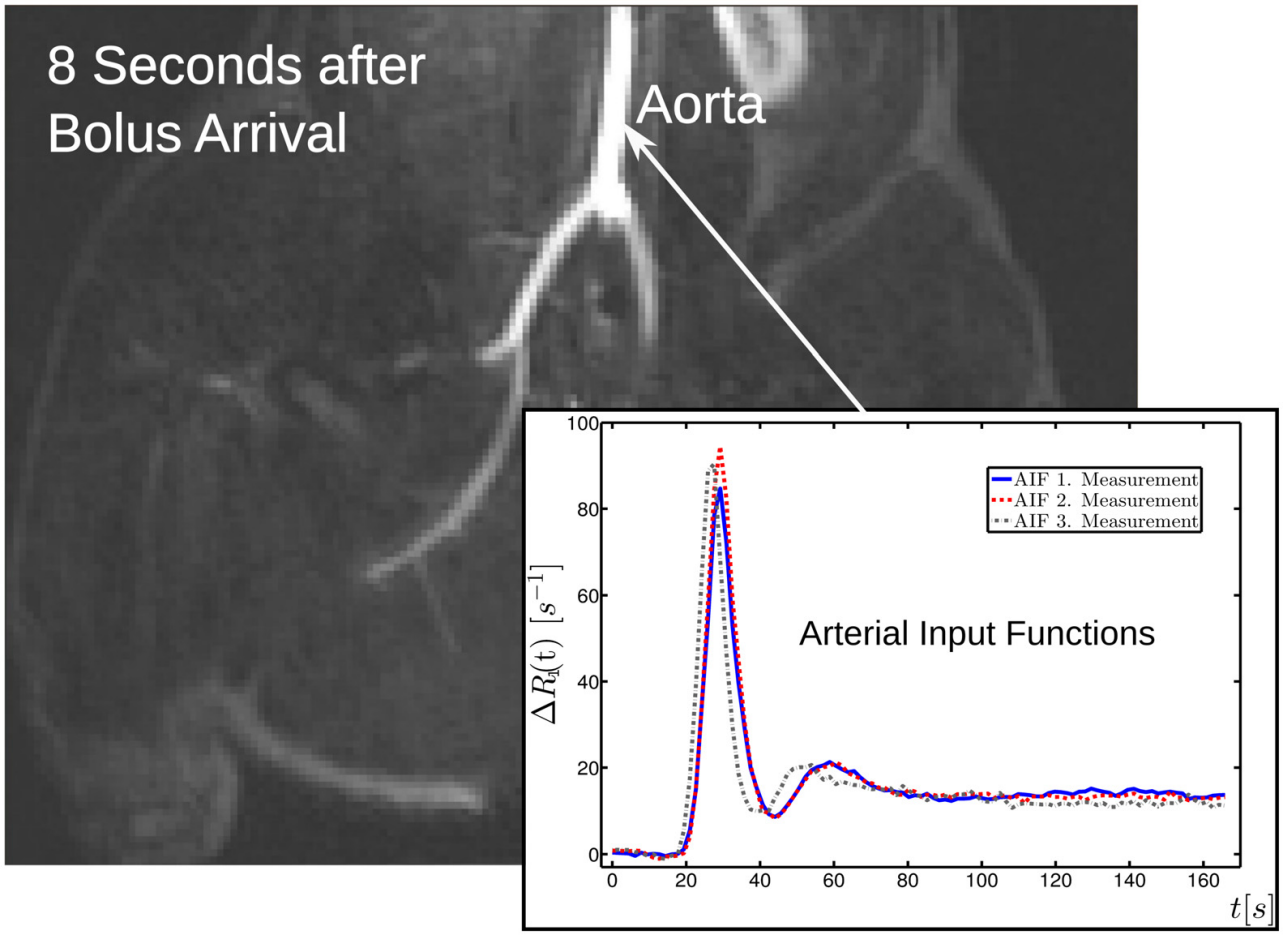
Coronal image of a dynamic GRE scan. Coronal image of a dynamic GRE scan obtained eight seconds after bolus arrival. Also shown are examples of the three arterial input function measurements in the abdominal aorta.

Seven animals were successfully examined and twenty flow measurements were done. The dynamic scans covered the entire abdomen including a large part of the aorta (Fig 6). To generate arterial input functions, we averaged over a set of about twenty full blood voxels in the aorta. The morphological and dynamic scans acquired with local CM administration (shown in Fig 2) were used to outline different muscle segments (see Fig 3). Apart from the displayed segments (cranial, lateral, medial thigh muscle, lower leg, skin, fat, and bone) the border region between lower leg and upper leg was outlined as an eighth region because the assignment to the respective segments was not clear enough in this intermediate area. However, for the flow calculation by multiplication of segment perfusion and segment volume followed by summation of the individual contributions, it was of crucial importance to include the entire hind leg volume.

Fig 3 presents examples of the tissue blood time curves of a single pig for three perfusion values and four different muscle segments (lateral and cranial thigh muscle, pelvic muscle and lower leg). In these examples, the lateral and cranial thigh muscle shows no response to administration of the vasodilator adenosine. In all experiments, the lower leg exhibited the strongest tissue reaction to adenosine administration. In this particular example, the pelvic muscle also shows a strong reaction. The response to local adenosine injection into the femoral artery resulted in an increase in the general height of the tissue curve and especially in a strong bolus increase in height and slope.

**Fig 7:**
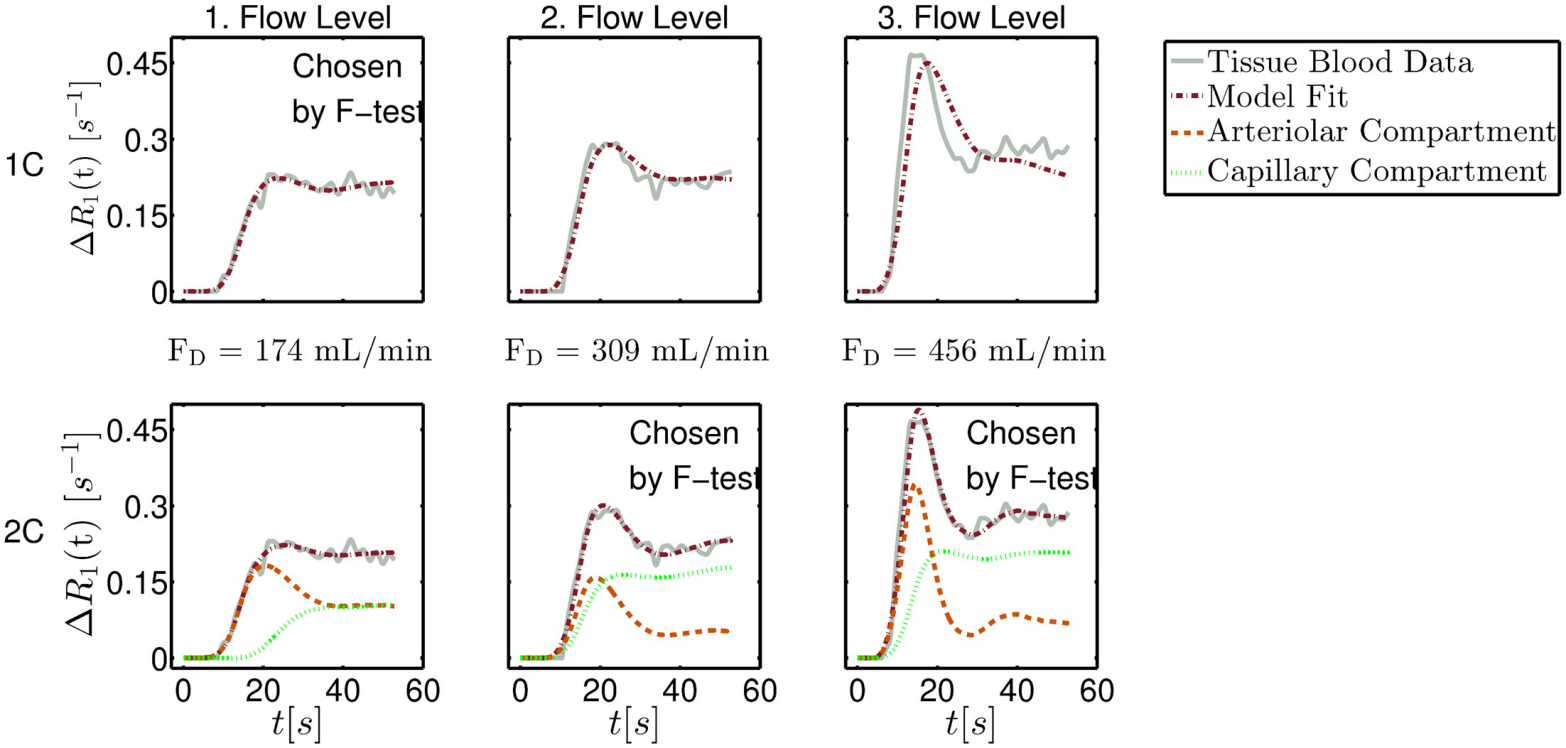
Fitting results for the two different models (one-compartment (1C) and two-compartment (2C)). Fitting results for the two different models (one-compartment (1C) and two-compartment (2C)) and for different flow levels. The solid line represents the tissue blood data and the dashed-dotted line represents the fitting result. The dotted (dashed) line shows the fitting result for the capillary (arteriolar) compartment.

Fitting examples for the one- and the two-compartment-model are presented in Fig 7. The examples show the fit to the tissue blood-time curves at three different stable flow levels in the lower leg segment. The F-test chooses the model fit with respect to the goodness of fit and the number of model parameters. For the first and lowest blood flow level (174 mL/min), the F-test chose the one-compartment model. For the second (309 mL/min) and third (456 mL/min) flow levels with significantly higher flows measured with the Doppler probe, and thus a high bolus contribution, the parallel two-compartment model was chosen by the F-test. Especially for the third measurement, with the large bolus contribution, the one-compartment model underestimates the bolus increase as well as the washout region.

**Fig 8:**
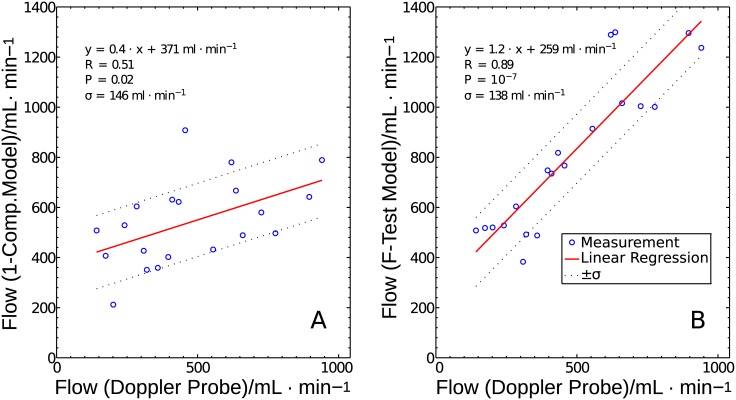
Regression of the corrected DCE-MRI models. Regression of the corrected DCE-MRI model results with the Doppler flow values for the one-compartment model (A) and for the F-test selection method (B). The solid line represents the linear regression fit of the measured data (circles). The dotted line depicts the standard deviation of the data related to the linear regression.

We plotted 20 flow values from seven pigs measured with the DCE-MRI model versus the corresponding Doppler flow values (Fig 8). If only the one-compartment model is used for all segments (Fig 8A), a correlation of *R* = 0.51 is obtained with a significance of *P* = 0.02, a slope of the regression line of *m* = 0.4, and an ordinate axis intercept of the regression line of 371 mL/min. Ideally, a slope of 1 and an ordinate intercept of zero would be expected. If we take into account both the one-compartment and the two-compartment model and let the F-test decide which one delivers the most accurate results (Fig 8B), we obtain a correlation of the flow values of *R* = 0.89 with a significance of *P* = 10^−7^, a slope of the regression line of 1.2, and an ordinate intercept of 259 mL/min. The ordinate intercept of 259 mL/min represents an average perfusion of the hind leg of 4.7 mL/(min⋅100cm^3^).

**Fig 9:**
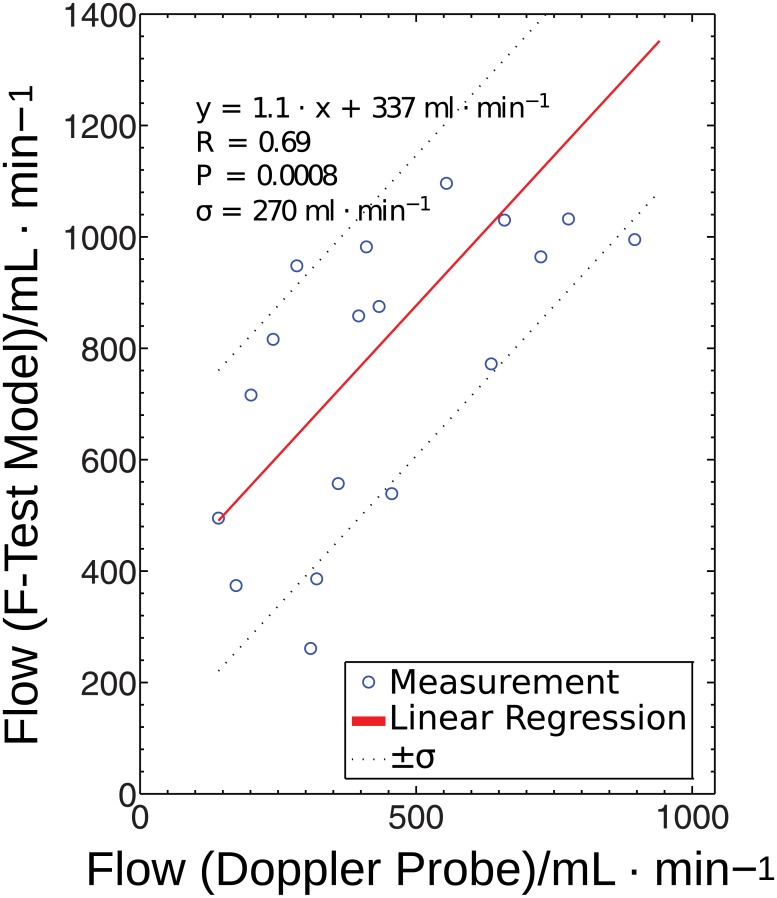
Regression of the uncorrected DCE-MRI model. Regression of the uncorrected DCE-MRI model results with the Doppler flow values for the F-test selection method. The solid line represents the linear regression fit of the measured data (circles). The dotted line depicts the standard deviation of the data related to the linear regression.

Fig 9 depicts the correlation of the uncorrected results obtained with the F-test selection method and the flow values measured with the Doppler probe. With a correlation of *R* = 0.69 and significance of *P* < 0.001, the correlation is still high. The slope of the regression line, *m* = 1.1, is very close to the expected result. However, the standard deviation *σ* is 270 mL/min for the uncorrected case and only 138 mL/min for the corrected case. Also, the ordinate intercept is higher (337 mL/min) than for the corrected case (259 mL/min). This result underlines that, in the present setting, flow correction with respect to the AIF steady state is justified.

**Fig 10:**
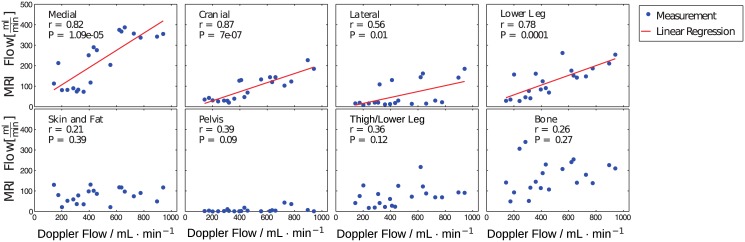
Correlation of the corrected DCE-MRI model results. Correlation of the corrected DCE-MRI model results with the Doppler flow values for the F-test selection method in the different muscle segments. The measurements are plotted using blue dots and the significant correlations (P<0.05) using a red line.

Table 1 presents mean perfusion values in the eight different muscle (values determined by using the F-test and corrected AIF) and tissue segments for four categories of flow values measured with the Doppler probe. The perfusion values measured with DCE-MRI ranged from 2 mL/(min⋅100 cm^3^) to up to 44 mL/(min⋅100 cm^3^). Moreover, especially in the muscle segments with low and moderate perfusion (Fig 10, upper row), there were high correlations between the corrected DCE-MR flow values and the Doppler flows, *R* = 0.56–0.87. The faintest response to adenosine administration was found in non-muscular tissue, namely bone, skin, and fat. The highest blood flow increase was observed in the medial thigh muscle (Fig 10, upper left plot).

**Table 1:** Mean perfusion values in four different Doppler flow regions and for the different muscle segments.

			Doppler Flow [mL/min]			
Segment		142–320	359–456	555–726	776–941
Lat. Thigh	Mean Perfusion [mL/(min⋅100cm^3^]	4	2	8	7
Cran. Thigh		3	9	21	14
Skin & Fat		8	11	10	12
Med. Thigh		8	14	23	25
Lower Leg		9	15	32	23
Pelvis		14	11	22	24
Thigh/L.Leg		19	18	31	44
Bone		23	25	27	29

Mean perfusion values in four different Doppler flow regions and for the different muscle segments.

For flow calculation, the entire perfused volume supplied by the femoral artery had to be included. Therefore, voxels not definitely assignable to the upper or lower leg had to be summarized in a separate segment referred to as “thigh/lower leg” and including contributions of skin and fat. Compared to the high correlations in the thigh muscles and in the lower leg, the low correlation in the combined segment confirms that segmentation of the muscles was necessary.

**Fig 11:**
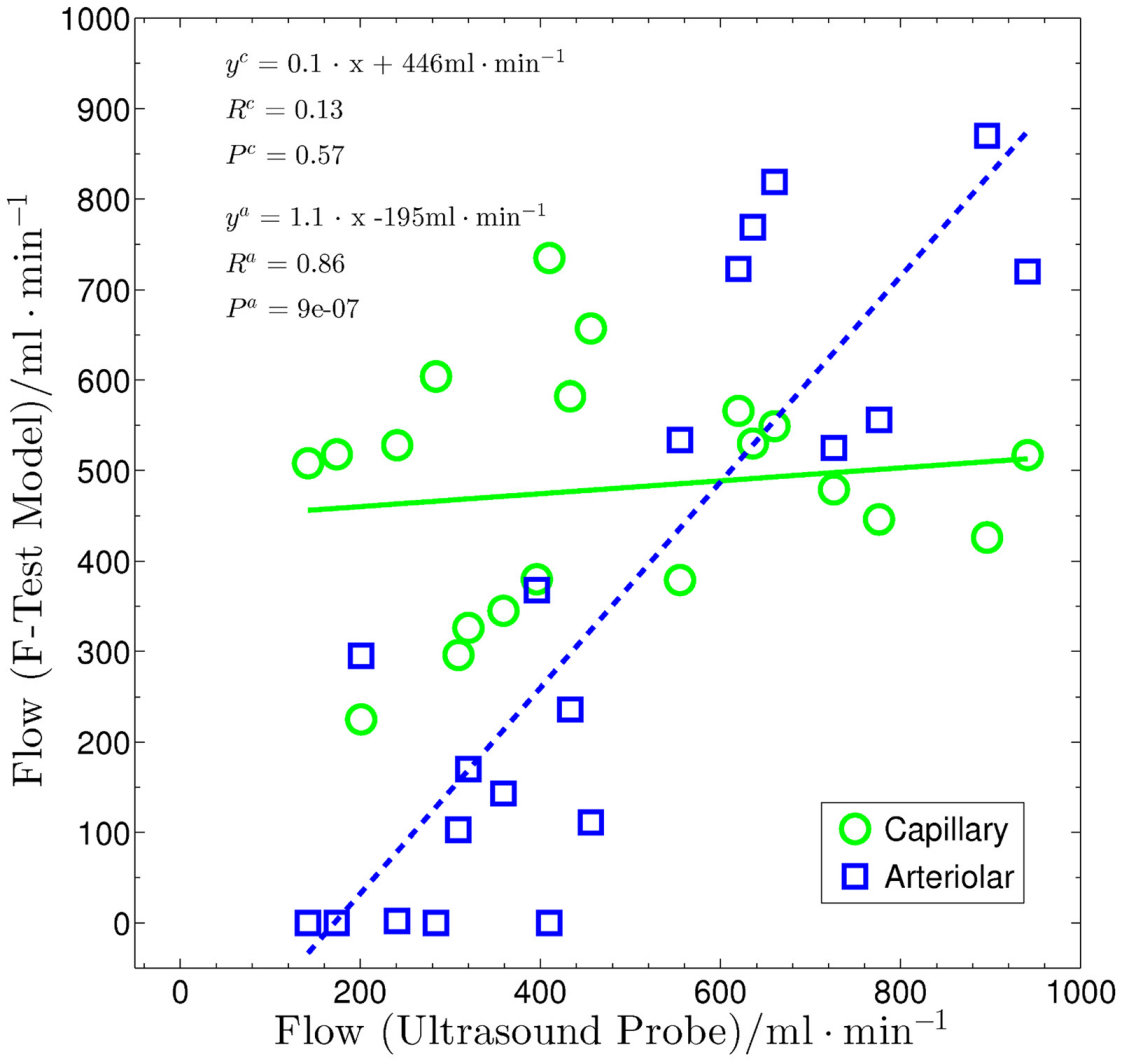
Regression of the corrected DCE-MRI model results. Regression of the corrected DCE-MRI model results with the Doppler flow values for the F-test selection method. The solid line represents the linear regression fit of the measured data with the capillary contribution of the model flow values (circles). The dashed line depicts the linear regression fit of the measured data with the arteriolar contribution of the model flow values (squares).

Plotting the arteriolar and capillary contributions of the corrected F-test model flows against the Doppler measurements (Fig 11) shows that the flow increase is mainly detected in the arteriolar compartment. The overall flow of the hind leg muscles, as measured by the ultrasound probe, highly correlates with the arteriolar contribution of the flow from the MRI measurement, *R* ^*a*^ = 0.86 and *P* ^*a*^ = 9 ⋅ 10^−7^. Linear regression yields a slope of 1.1 and a y-axis intercept of -195 mL/min. The capillary flow contribution of the MRI measurement results in a correlation coefficient of only *R* ^*c*^ = 0.13 with a significance of *P* ^*c*^ = 0.57, and linear regression reveals a slope of 0.1 and a y-axis intercept of 446 mL/min. These results suggest that flow increases caused by adenosine administration are mainly due to the activation of arterio-venous shunts [32–36].

## Discussion

In our experiments, dynamic scans were acquired using a k-space undersampling and data-sharing method known as Time-resolved angiography With Stochastic Trajectories (TWIST) [37, 38]. This technique yields sufficiently high temporal resolution without sacrificing spatial resolution and anatomic coverage. However, k-space undersampling may distort enhancement curves, especially when the signal is changing rapidly, for example, during first-pass perfusion [37]. This might affect the accuracy of our kinetic modeling parameters especially with regard to the determination of the arterial input function and the generation of high-flow tissue blood curves with a large bolus contribution, where the two-compartment model is employed. A central region of 20% and a sampling density of about 20% is often used in the tracer kinetic community [37, 39]. This approach combines high temporal resolution of approx. 1.5 seconds with minimal view sharing of the non-bolus phases during first pass (full width at half maximum (FWHM) ≈ 10 seconds).

However, the inclusion of k-space lines from the surrounding time points may lead to a drop of the bolus peak. A too low AIF bolus peak leads to perfusion overestimation. This holds in particular for cases in which the F-test selects the arteriolar compartment for flow states with a large bolus contribution (Fig 7 right plot column). Thus, especially at high flow states, the TWIST technique may overestimate flow. This may explain a slope of the regression line larger than 1 and/or the y-axis intersection in the correlation plot (Fig 8B). Since tumors can be very small (few mm), the small number of k-lines sampled every time point also might be a hindrance in actual cinical applications. Moreover, since tumors tend to have leaky vasculature, the intravascular agent will leak into the interstitial compartment thereby producing the condition that would exist in tissue with intact vasculature and use of a low-molecular-weight contrast agent that passes into the interstitial compartment. However, in this study we want to restrict the analysis to the general case of healthy tissue as a basis for more advanced applications.

Incorrect bolus dispersion times might led to underestimation or overestimation of dispersion for the arteriolar compartment. Compared to the actual time resolution, the dispersion times are rather small. Along with the inherent errors of the shared k-space technique, this might preclude determination of dispersion times altogether.

Kershaw et al. suggest that an accurate measurement of model parameters relies on acquiring data with high temporal resolution and low noise, particularly for models with large numbers of free parameters [40]. They investigated model accuracy in a simulation study, examining the effects of temporal resolution, noise levels, and error on the measured arterial input function. A temporal resolution of 1.5 s and high signal-to-noise ratio (SNR; noise sd = 0.05) were found to ensure minimal bias (< 5%) in all four model parameters (in their study: extraction fraction, blood flow, mean transit time, and extravascular extracellular volume).

Temporal resolution in our experiments was approximately 1.5 s. We calculated a contrast-to-noise ratio (CNR), by dividing the average height of the T1 relaxation rate change time curves by the standard deviation of the time curves in the ten seconds before bolus arrival. The CNR of the tissue blood curves was enhanced by averaging over all voxels of the muscle segments. Even for the lowest blood tissue uptake curves, we obtained CNRs of approximately 9 (noise SD = 0.04). Because of the height of the arterial input signal of the aorta we had a CNR of the arterial input function in the order of 70, but due to averaging over just up to twenty full blood voxels, we had a rather high arterial input noise SD of 0.25.

Despite the administration of the same body weight-adjusted CM dose in all pigs, there were significant differences among the seven experiments in the height of the steady-state phase of the AIFs. However, the steady-state phases of the three AIF measurements within one experiment had approximately the same height (see Fig 6). The signal differences may be attributable to magnetic field inhomogeneities in the few AIF voxels at the edge of the TIM body coil and differences in automated shimming between the seven experiments.

In addition, we cannot rule out that, in the coil border regions, the true flip angles deviated from the prescribed sequence values. When converting the scanner signals into a relaxation rate change map, this effect will naturally lead to systematic errors. Moreover, accuracy of signal calculation is expected to be highest in the coil center since each coil element of the body coil has a maximum number of neighbors. Therefore, in the border region of the coil (where the abdominal aorta AIF is measured), signal corruption may take place.

Moreover, the amount of inflowing spins depended on the heart rate of each pig at the time of measurement. Thus, different rates of new inflowing spins not in steady state may lead to differences between the various AIF heights. The AIF had approximately 100 times higher signal values than the tissue blood curves, causing errors in the neighboring AIF voxels to be more heavily weighted. Neglecting T2* effects could be an additional source of error.

Data quality might have decreased slightly from the first to the third measurement due to contrast medium saturation, thus causing a lower CNR. However, significant data degradation was not apparent. It is conceivable to take four measurements in future experiments. However, to reduce a potential impact on flow correlation, we varied the order of the flow level change within each experiment.

The accuracy of the reference Doppler flow probe is, according to manufacturer specifications, ±10% [41]. Due to interference by the scanner signal, flow could not be measured by the Doppler flow meter during MRI acquisitions. Therefore, Doppler flow values obtained right before the start of MR scans were averaged. The flow levels were stable for five minutes before starting the MR measurement, and therefore significant drifts in flow levels during imaging were unlikely.

### Perfusion Modeling

Use of a more complex model does not automatically result in higher accuracy or precision. Fitting a model is a reasonable method to learn more about underlying tissue physiology. But if too few parameters are used, the model does not describe the data (underfitting). On the other hand, using too many parameters may lead to a situation where the extra parameters only describe noise (overfitting). Our guiding principle was that the simplest model that describes the data is the most appropriate. If a set of models with different numbers of parameters is used, then the appropriate model for fitting the data can be determined by using statistical tests for model comparison.

It is not clear whether the compartments actually separate physiological information. For example, we cannot say with certainty that what we measure in the arteriolar compartment actually describes physiologic information gathered solely from the tissue arteries and arterioles. At least, with better separation of different physiological structures, the capability of the model to provide information on these separate structures increases. While the delay and dispersion correction of the individual arterial input functions improves separation of the contributions from the two compartments, the use of more model parameters leads to additional problems such as overfitting, the same curve can be described by different parameter sets. As a result, it is difficult to separate the effects of the vascular transport function from those of the residual function resulting, in under- or overestimation of perfusion.

Comparison of the flow results using only the one-compartment model versus F-test selection (Fig 8) clearly shows that the combined use of both models is necessary for accurate perfusion determination. Exclusive use of the two-compartment model leads to a correlation between MRI flow values and Doppler flows of *R* = 0.66, with a significance of *P* < 0.002, a regression line of *y* = 1.2*x* + 689 mL/min, and a standard deviation from the regression line of 335 mL/min. Thus, the two-compartment model improves determination of the flow increase compared with the one-compartment model. F-test selection prevents curve fitting improvement due to noise rather than physiologically accessible information and thus provides the most accurate overall results. However, as stated by Sourbron et al. in [30], automated selection criteria such as the F-test are useful as indicators for global trends, but they are not reliable on a case-by-case basis, or when differences are more subtle. Therefore, due to incorrect selections by the F-test in individual cases, statistical errors may arise.

Our results prove that, with a large amount of data and use of delay and dispersion correction, it is possible to obtain precise flow measurements even in low-perfusion tissue. To obtain precise results, it is most important to use different models for high and low tissue perfusion states and to use a statistical test to choose the optimal model for each case (as suggested by [30] and [31]). Our results in Fig 8 and the results using only the two-compartment model demonstrate that the two-compartment model overestimates flow in tissue in which no structural separation is possible between bolus phase and washout. Applying the one-compartment model in regions where this separation is possible will most likely result in an underestimation of flow.

A drawback of the use of delay and dispersion correction is that additional model parameters must be taken into account. According to Sourbron and Buckley [17], the state of an n-compartment model can only be identified when the corresponding residue function contains n clearly separated exponential terms. Hence, to stabilize the fit and to restrict the parameter space of the model, physically meaningful constraints were imposed on parameters. For example, it was required that the bolus delay and the bolus dispersion of the arteriolar compartment should always be lower than those of the capillary compartment.

The perfusion values measured in the present study cover the range of flow levels reported in the literature for low-flow muscle, healthy and diseased prostate, and the lower end of high muscle perfusion during activity. Thus, our study shows that muscle blood flow can be used as a tissue model for perfusion validation studies. Armstrong et al. determined average muscle perfusion of the hind leg of miniature pigs during treadmills exercises. Depending on stress, the values ranged between 20 and 124 mL/(min⋅cm^3^) [42]. Moreover, our results confirm several earlier studies [15, 42–46] stating that muscle perfusion differs among muscles and that primarily the high-oxidative extensor muscles have blood flows above average muscle blood flow. Furthermore, our study confirms that muscles have the ability to adapt their perfusion to physiologic requirements.

Moreover, whereas normal microvasculature is characterized by well structured branching, tumor angiogenesis in prostate cancer is characterized morphologically by an increase in the number of blood vessels including new capillaries, capillary sprouts, non-endothelialized capillaries and arterio-venous shunts [32–36, 47–49]. Different vessel types might also significantly contribute to local perfusion as well as found in the muscle. Thus, the findings of the present study might help in improving the accuracy of noninvasive imaging-based perfusion measurement in low-flow tumor tissue.

## Conclusion

The DCE-MRI technique presented here uses a blood pool contrast agent and a F-test selection method for different model approaches. It allows absolute quantification of skeletal muscle flow and is thus applicable for low perfused organs like prostate and breast. Our results show that the technique has sufficient accuracy and reproducibility to be transfered to the clinical setting.

## References

[1] Gilkes DM, Semenza GL (2013) Role of hypoxia-inducible factors in breast cancer metastasis. Future oncology (London, England) 9: 1623–1636. 10.2217/fon.13.92 PMC4659402 24156323

[2] Jordan BF, Sonveaux P (2012) Targeting tumor perfusion and oxygenation to improve the outcome of anticancer therapy. Frontiers in pharmacology 3: 94 10.3389/fphar.2012.00094 22661950 PMC3357106

[3] Gillies RJ, Schornack PA, Secomb TW, Raghunand N (1999) Causes and effects of heterogeneous perfusion in tumors. Neoplasia (New York, NY) 1: 197–207. 10.1038/sj.neo.7900037 PMC1508079 10935474

[4] Höckel M, Vaupel P (2001) Tumor hypoxia: definitions and current clinical, biologic, and molecular aspects. Journal of the National Cancer Institute 93: 266–276. 10.1093/jnci/93.4.266 11181773

[5] Stewart B, Wild C (2014) World Cancer Report 2014 International Agency for Research on Cancer. World Health Organization.

[6] Chaudary N, Hill RP (2006) Hypoxia and metastasis in breast cancer. Breast disease 26: 55–64. 17473365 10.3233/bd-2007-26105

[7] Baade PD, Youlden DR, Krnjacki LJ (2009) International epidemiology of prostate cancer: geographical distribution and secular trends. Mol Nutr Food Res 53: 171–184. 10.1002/mnfr.200700511 19101947

[8] Budach V, Stuschke M, Budach W, Baumann M, Geismar D, et al (2005) Hyperfractionated accelerated chemoradiation with concurrent fluorouracil-mitomycin is more effective than dose-escalated hyperfractionated accelerated radiation therapy alone in locally advanced head and neck cancer: final results of the radiotherapy cooperative clinical trials group of the german cancer society 95–06 prospective randomized trial. J Clin Oncol 23: 1125–1135. 15718308 10.1200/JCO.2005.07.010

[9] Brizel DM, Albers ME, Fisher SR, Scher RL, Richtsmeier WJ, et al (1998) Hyperfractionated irradiation with or without concurrent chemotherapy for locally advanced head and neck cancer. N Engl J Med 338: 1798–1804. 10.1056/NEJM199806183382503 9632446

[10] Vaupel P, Kelleher DK (2013) Blood flow and oxygenation status of prostate cancers. Advances in experimental medicine and biology 765: 299–305. 22879048 10.1007/978-1-4614-4989-8_42

[11] Nahum AE, Movsas B, Horwitz EM, Stobbe CC, Chapman JD (2003) Incorporating clinical measurements of hypoxia into tumor local control modeling of prostate cancer: implications for the alpha/beta ratio. Int J Radiat Oncol Biol Phys 57: 391–401. 10.1016/S0360-3016(03)00534-0 12957250

[12] Vaupel P (1990) Oxygenation of human tumors. Strahlentherapie und Onkologie: Organ der Deutschen Rontgengesellschaft [et al] 166: 377–386. 2194302

[13] Urbanchek MG, Picken EB, Kalliainen LK, Kuzon WM (2001) Specific force deficit in skeletal muscles of old rats is partially explained by the existence of denervated muscle fibers. The journals of gerontology Series A, Biological sciences and medical sciences 56: B191–B197. 10.1093/gerona/56.5.B191 11320099

[14] Wilson CB, Lammertsma AA, McKenzie CG, Sikora K, Jones T (1992) Measurements of blood flow and exchanging water space in breast tumors using positron emission tomography: a rapid and noninvasive dynamic method. Cancer research 52: 1592–1597. 1540969

[15] Laughlin MH (1987) Skeletal muscle blood flow capacity: role of muscle pump in exercise hyperemia. The American journal of physiology 253: H993–1004. 3318504 10.1152/ajpheart.1987.253.5.H993

[16] Durán WN, Renkin EM (1974) Oxygen consumption and blood flow in resting mammalian skeletal muscle. The American journal of physiology 226: 173–177. 4809879 10.1152/ajplegacy.1974.226.1.173

[17] Sourbron SP, Buckley DL (2013) Classic models for dynamic contrast-enhanced mri. NMR in biomedicine 26: 1004–1027. 10.1002/nbm.2940 23674304

[18] Lüdemann L, Nafz B, Elsner F, Grosse-Siestrup C, Meissler M, et al (2009) Absolute quantification of regional renal blood flow in swine by dynamic contrast-enhanced magnetic resonance imaging using a blood pool contrast agent. Investigative radiology 44: 125–134. 10.1097/RLI.0b013e318193598c 19151609

[19] Lüdemann L, Prochnow D, Rohlfing T, Franiel T, Warmuth C, et al (2009) Simultaneous quantification of perfusion and permeability in the prostate using dynamic contrast-enhanced magnetic resonance imaging with an inversion-prepared dual-contrast sequence. Annals of biomedical engineering 37: 749–762. 10.1007/s10439-009-9645-x 19169821

[20] Calamante F, Gadian DG, Connelly A (2000) Delay and dispersion effects in dynamic susceptibility contrast mri: simulations using singular value decomposition. Magnetic resonance in medicine: official journal of the Society of Magnetic Resonance in Medicine / Society of Magnetic Resonance in Medicine 44: 466–473. 10.1002/1522-2594(200009)44:3<466::AID-MRM18>3.3.CO;2-D 10975900

[21] Calamante F, Willats L, Gadian DG, Connelly A (2006) Bolus delay and dispersion in perfusion mri: implications for tissue predictor models in stroke. Magnetic resonance in medicine: official journal of the Society of Magnetic Resonance in Medicine / Society of Magnetic Resonance in Medicine 55: 1180–1185. 10.1002/mrm.20873 16598717

[22] Ibaraki M, Shimosegawa E, Toyoshima H, Ishigame K, Ito H, et al (2005) Effect of regional tracer delay on cbf in healthy subjects measured with dynamic susceptibility contrast-enhanced mri: comparison with 15o-pet. Magnetic resonance in medical sciences: MRMS: an official journal of Japan Society of Magnetic Resonance in Medicine 4: 27–34. 10.2463/mrms.4.27 16127251

[23] Ibaraki M, Shimosegawa E, Toyoshima H, Takahashi K, Miura S, et al (2005) Tracer delay correction of cerebral blood flow with dynamic susceptibility contrast-enhanced mri. Journal of cerebral blood flow and metabolism: official journal of the International Society of Cerebral Blood Flow and Metabolism 25: 378–390. 10.1038/sj.jcbfm.9600037 15674238

[24] Sauerbrey A, Hindel S, Maaß M, Krüger C, Wissmann A, et al (2014) Establishment of a swine model for validation of perfusion measurement by dynamic contrast-enhanced magnetic resonance imaging. BioMed research international 2014: 390506 10.1155/2014/390506 24719859 PMC3955654

[25] Li KL, Zhu XP, Waterton J, Jackson A (2000) Improved 3d quantitative mapping of blood volume and endothelial permeability in brain tumors. Journal of magnetic resonance imaging: JMRI 12: 347–357. 10.1002/1522-2586(200008)12:2<347::AID-JMRI19>3.0.CO;2-7 10931600

[26] Rohrer M, Bauer H, Mintorovitch J, Requardt M, Weinmann HJ (2005) Comparison of magnetic properties of MRI contrast media solutions at different magnetic field strengths. Invest Radiol 40: 715–724. 10.1097/01.rli.0000184756.66360.d3 16230904

[27] Ostergaard L, Weisskoff RM, Chesler DA, Gyldensted C, Rosen BR (1996) High resolution measurement of cerebral blood flow using intravascular tracer bolus passages. part i: Mathematical approach and statistical analysis. Magnetic resonance in medicine: official journal of the Society of Magnetic Resonance in Medicine / Society of Magnetic Resonance in Medicine 36: 715–725. 10.1002/mrm.1910360510 8916022

[28] Tofts PS, Brix G, Buckley DL, Evelhoch JL, Henderson E, et al (1999) Estimating kinetic parameters from dynamic contrast-enhanced t(1)-weighted mri of a diffusable tracer: standardized quantities and symbols. Journal of magnetic resonance imaging: JMRI 10: 223–232. 10.1002/(SICI)1522-2586(199909)10:3<223::AID-JMRI2>3.0.CO;2-S 10508281

[29] Renkin E (1984) Handbook of physiology: Section 2, The cardiovascular system. Microcirculation: pt. 2. Bd. 4. American Physiological Soc.

[30] Sourbron SP, Buckley DL (2012) Tracer kinetic modelling in mri: estimating perfusion and capillary permeability. Physics in medicine and biology 57: R1–33. 10.1088/0031-9155/57/2/R1 22173205

[31] Donaldson SB, West CML, Davidson SE, Carrington BM, Hutchison G, et al (2010) A comparison of tracer kinetic models for t1-weighted dynamic contrast-enhanced mri: Application in carcinoma of the cervix. Magnetic Resonance in Medicine 63: 691–700. 10.1002/mrm.22217 20187179

[32] Molls M, Vaupel P, Nieder C, Anscher M (2009) The Impact of Tumor Biology on Cancer Treatment and Multidisciplinary Strategies. 1 st ed Springer-Verlag Berlin Heidelberg.

[33] Vaupel P, Grunewald W, Manz R, Sowa W (1978) Intracapillary hbo2 saturation in tumor tissue of ds-carcinosarcoma during normoxia. Adv Exp Med Biol 94: 367–375. 10.1007/978-1-4684-8890-6_48 613778

[34] Weiss L, Hultborn R, Tveit E (1979) Blood flow characteristics in induced rat mammary neoplasia. Microvasc Res 17: 119.

[35] Endrich B, Hammersen F, Goetz A, Messmer K (1982) Microcirculatory blood flow, capillary morphology, and local oxygen pressure of the hamster amelanotic melanoma a-mel-3. J Natl Cancer Inst 68: 475–485. 6950176

[36] Wheeler R, Ziessman H, Medvec B, Juni J, Thrall J, et al (1986) Tumor blood flow and systemic shunting in patients receiving intra-arterial chemotherapy for head and neck cancer. Cancer Res 46: 4200–4204. 3488122

[37] Song T, Laine AF, Chen Q, Rusinek H, Bokacheva L, et al (2009) Optimal k-space sampling for dynamic contrast-enhanced mri with an application to mr renography. Magnetic resonance in medicine: official journal of the Society of Magnetic Resonance in Medicine / Society of Magnetic Resonance in Medicine 61: 1242–1248. 10.1002/mrm.21901 PMC2773550 19230014

[38] Laub G, Kroeker R (2006) Syngo twist for dynamic time-resolved mr angiography. MAGNETOM FLASH.

[39] Sourbron S, Sommer WH, Reiser MF, Zech CJ (2012) Combined quantification of liver perfusion and function with dynamic gadoxetic acid-enhanced mr imaging. Radiology 263: 874–883. 10.1148/radiol.12110337 22623698

[40] Kershaw LE, Cheng HLM (2010) Temporal resolution and snr requirements for accurate dce-mri data analysis using the aath model. Magnetic resonance in medicine: official journal of the Society of Magnetic Resonance in Medicine / Society of Magnetic Resonance in Medicine 64: 1772–1780. 10.1002/mrm.22573 20715059

[41] Transonic Animal Research Flowmeters T106/T206 Series.

[42] Armstrong RB, Delp MD, Goljan EF, Laughlin MH (1987) Distribution of blood flow in muscles of miniature swine during exercise. Journal of applied physiology (Bethesda, Md: 1985) 62: 1285–1298. 10.1152/jappl.1987.62.3.1285 3106313

[43] Laughlin MH, Armstrong RB (1982) Muscular blood flow distribution patterns as a function of running speed in rats. The American journal of physiology 243: H296–H306. 7114239 10.1152/ajpheart.1982.243.2.H296

[44] Laughlin MH, Mohrman SJ, Armstrong RB (1984) Muscular blood flow distribution patterns in the hindlimb of swimming rats. The American journal of physiology 246: H398–H403. 6703075 10.1152/ajpheart.1984.246.3.H398

[45] Laughlin MH, Armstrong RB (1985) Muscle blood flow during locomotory exercise. Exercise and sport sciences reviews 13: 95–136. 10.1249/00003677-198500130-00006 3891377

[46] Armstrong RB, Laughlin MH (1985) Rat muscle blood flows during high-speed locomotion. Journal of applied physiology (Bethesda, Md: 1985) 59: 1322–1328. 10.1152/jappl.1985.59.4.1322 4055609

[47] Luczyńska E, Anioł J (2013) Neoangiogenesis in prostate cancer. Contemporary oncology (Poznań, Poland) 17: 229–233. 10.5114/wo.2013.35272 PMC3934077 24596506

[48] Russo G, Mischi M, Scheepens W, De la Rosette JJ, Wijkstra H (2012) Angiogenesis in prostate cancer: onset, progression and imaging. BJU Int 110: 794–808. 10.1111/j.1464-410X.2012.11444.x 22958524

[49] Mork C, Asker CL, Salerud EG, Kvernebo K (2000) Microvascular arteriovenous shunting is a probable pathogenetic mechanism in erythromelalgia. The Journal of investigative dermatology 114: 643–646. 10.1046/j.1523-1747.2000.00944.x 10733667

